# Controlled Deposition of Nanostructured Hierarchical TiO_2_ Thin Films by Low Pressure Supersonic Plasma Jets

**DOI:** 10.3390/nano12030533

**Published:** 2022-02-03

**Authors:** Cecilia Piferi, Chiara Carra, Kateryna Bazaka, Hector Eduardo Roman, Elisa Camilla Dell’Orto, Vittorio Morandi, Igor Levchenko, Claudia Riccardi

**Affiliations:** 1 Department of Physics, University of Milano-Bicocca, Piazza della Scienza 3, 20126 Milan, Italy; c.piferi@campus.unimib.it (C.P.); c.carra@campus.unimib.it (C.C.); eduardo.roman@mib.infn.it (H.E.R.); elisa.dellorto@unimib.it (E.C.D.); 2 School of Engineering, College of Engineering and Computer Science, The Australian National University, Canberra 2600, Australia; katia.bazaka@anu.edu.au; 3 CNR-IMM Sede di Bologna, Via Gobetti 101, 40129 Bologna, Italy; morandi@bo.imm.cnr.it; 4 Plasma Sources and Applications Centre, NIE, Nanyang Technological University, Singapore 637616, Singapore; levchenko.igor@nie.edu.sg

**Keywords:** plasma, supersonic jet, deposition, nanostructures, TiO_2_ film

## Abstract

Plasma-assisted supersonic jet deposition (PA-SJD) is a precise technique for the fabrication of thin films with a desired nanostructured morphology. In this work, we used quadrupole mass spectrometry of the neutral species in the jet and the extensive characterization of TiO_2_ films to improve our understanding of the relationship between jet chemistry and film properties. To do this, an organo–metallic precursor (titanium tetra–isopropoxide or TTIP) was first dissociated using a reactive argon–oxygen plasma in a vacuum chamber and then delivered into a second, lower pressure chamber through a nozzle. The pressure difference between the two chambers generated a supersonic jet carrying nanoparticles of TiO_2_ in the second chamber, and these were deposited onto the surface of a substrate located few centimeters away from the nozzle. The nucleation/aggregation of the jet nanoparticles could be accurately tuned by a suitable choice of control parameters in order to produce the required structures. We demonstrate that high-quality films of up to several µm in thickness and covering a surface area of few cm^2^ can be effectively produced using this PA-SJD technique.

## 1. Introduction

The demand for new materials with specific features at nano/micro-scales has been considerably increasing in recent times. This is a consequence of the superior and/or unusual properties that arise from the unique combination of chemistry and complex structure across multiple length scales. Nanoparticles (NPs) are often the building blocks of choice for the synthesis and assembly of advanced nanomaterials with high chemical reactivity and mechanical strength, as well as new optical and electrical properties. This is because of their wide range of available chemistries, different shapes of varying sizes, large surface-to-bulk ratios, and quantum-confinement effects at small length scales. Metallic and metal oxide nanoparticles and their thin films play particularly important roles in the advancement [1,2] of sensors [3,4,5], photocatalysts [6,7], bio-electronic-based devices [8,9], super-hydrophobic and self-cleaning materials [10,11], and various nanoelectronic devices [12,13]. In addition, light-sensitive diamond films have found applications in space technology [14], while other types of nanostructured aggregates are promising for the achievement of propulsion systems based on plasma technologies [15,16]. Both space applications are envisaged as critical for the advancement of affordable and miniaturized space assets [17].

The quality, possible properties, and morphology of metal-oxide nanoparticles and their assemblies are defined to a large extent by the opportunities and limitations of the processes used for their synthesis and assembly, of which there are many [18,19,20]. For the synthesis of ordered structures with engineered pore architecture, methods that rely on templating agents are frequently used, with soft templates offering the advantage of lower costs, simpler preparation, and greater versatility. For instance, mesoporous materials composed of TiO_2_ microparticles have been produced by combining material precursors and block polymer micelles [21]. The template-free fabrication of ordered TiO_2_ nanostructured materials is also being actively investigated in an effort to devise a method that would enable a greater level of tunability of all material characteristics in a controlled and predictable manner. Indeed, techniques aimed at the production of thin films, such as physical vapor deposition (PVD) [22], plasma-enhanced chemical vapor deposition (PECVD) [23,24], and reactive pulsed laser deposition (PLD) [25,26] have had much success in realizing this objective. Another relevant technique is atomic layer deposition (ALD), which is nowadays one of the most versatile and controllable techniques for thin film deposition [27,28]. The more recent development of plasma-assisted supersonic jet deposition (PA-SJD) [29,30,31,32] may offer a greater level of both flexibility and control. This can be obtained by segmenting the synthesis of the new material from the input gas into two separate steps, thus allowing for a greater control of, first, the chemistry involved by fine-tuning the properties of the reactive cold plasma environment, and second, the nucleation and assembly of nanoparticles carried by the supersonic jet and impacting the substrate [30,33]. In addition to the enhanced control and versatility of the deposition technique, large deposition areas and high deposition rates may be realized, the latter obtained by using high density plasmas of volatile and stable precursors for oxides, semiconductors, or metals. Importantly, when using supersonic jet deposition, new types of sintering processes may emerge during deposition at or close to room temperature [34]. These features, coupled with the strong adsorption and electrical surface properties of thus-fabricated hierarchical nano-assemblies, make this process suitable for many technological applications [35,36,37,38,39,40,41].

This paper presents an exploration of the relationship between the characteristics of the supersonic jet and the hierarchical nano/micro-structural properties of the resulting thin films in detail. The plasma and deposition chambers used in PA-SJD play different roles in the process. The former is used to create nanoparticles of a uniform size. The latter determines the morphology of the deposited thin films depending on the plasma control parameters, i.e., by varying the size of the nozzle connecting both chambers and the kinetic energy of the nanoparticles transported by the supersonic jet. In this way, it is possible to obtain films with a wide range of structures, from tree-like structures with different openings to more compact ones resembling cauliflower fractal-like configurations, from the very same nanoparticle building blocks, the nature of which remains virtually unaltered due to their negligible interactions in the jet.

Along with the high deposition rate, the proposed technique has the advantage of being able to control both the morphology and the stoichiometry of the films with high accuracy due to the specifics of the plasma-based process. Although the process is carried out at low pressures, the deposition rates are quite high due to the benefits of the plasma environment.

Our technique ensures results similar to those obtainable with Pulsed Laser Deposition (PLD), but it has several advantages over PLD and similar techniques, namely:

(1) The process occurs at a low pressure that it is still higher than pressures used in PLD, and deposition at a room temperature promotes a higher level of scalability. All low-pressure systems have inherent challenges regarding integration and scale-up, but they also features definitive advantages. To overcome the scalability problem, several approaches are currently being explored, including designing a plasma source of larger volume to increase the surface area of films, using deposition systems that employ supersonic multi-jet systems, using systems with moving stages, and using other systems that could be adapted to the industrial scale.
(2) The stoichiometric control of film composition with a high degree of reliability independently on the deposition process by injecting a metallic organic precursor into a mixture with inert gases and oxygen is also an advantage. Compared to sputtering or PLD based on the ablation of a material, ‘direct’ plasmochemistry enables variance in a wider spectrum the composition of the metallic oxide film and the deposition of layers of different chemical compositions, thus realizing, for instance, the additive manufacturing of multi-metal-based materials.
(3) The direct control of the morphology of the deposited structures, by means of the compression ratio R acting on the pressure in the deposition chamber without therefore acting on the density of the precursor, allows researchers to create films with variable porosity and morphology, without the need to vary the nanoparticle size, in a well-controlled way (a fine morphology tuning).

These advantages are demonstrated in part in the paper. The question of changing the properties of the particles produced in the first chamber requires further investigation that is beyond the scope of the present work.

## 2. Experimental Setup

### 2.1. Plasma-Assisted Supersonic Jet Equipment

**Figure 1:**
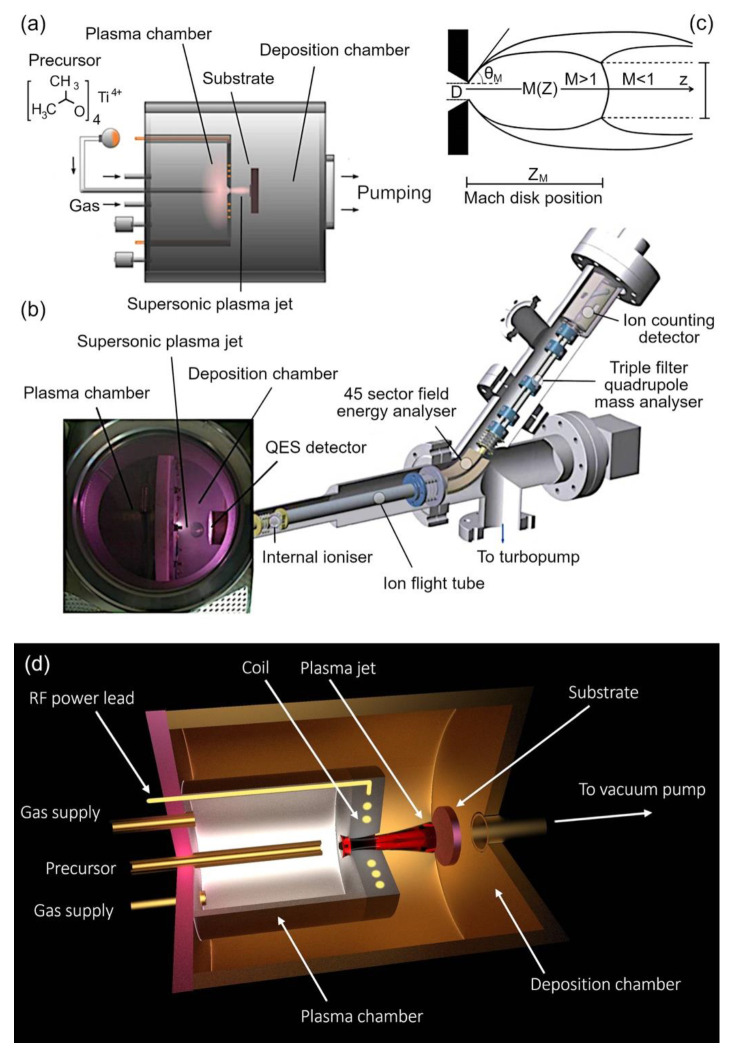
(**a**) Schematic representation of the experimental device. The whole vacuum vessel comprised two well-separated chambers. The positions of the RF coil antenna, nozzle, pumping system, the gas and precursor injections, and substrate holder are shown. (**b**) Photo of the PA-SJD: (left part) Plasma chamber, deposition chamber, and substrate holder right to the plasma jet; (right part) photo of the QMS detector used for the analysis of the plasma jet inside the deposition chamber. The QMS replaced the substrate holder during the mass measurements. (**c**) Scheme of the gas expansion in the chamber. The different Mach numbers, *M*, corresponding to the supersonic and subsonic regimes are indicated together with the position of the Mach disk at $z_{M}$ separating them. The jet was free to expand along both the axial and the radial directions before it reached the Mach disk, where the transition to the subsonic condition took place. The position of the sampling holder could be changed along the z-axis in a continuous fashion. (**d**) 3D rendering of the device.

The PA-SJD [42,43] technique consists of a two-step process confined within two stainless steel vacuum chambers of cylindrical shape that are connected via a small nozzle of varying aperture size. The plasma chamber used in this study had a length L_p_ = 95 mm and radius R_p_ = 62.5 mm, and the respective metrics of the deposition chamber were L_D_ = 200 mm and R_D_ = 160 mm (see Figure 1). At the bottom of the former, a circular conduit of typically 100 mm in diameter (which could be varied by adjusting a gate valve) connected the vessel to the main pumping system, which consisted of a turbo molecular and a rotary pump. In this study, the effective pumping speed was about 130 l/s for the pressures of 8 Pa in the plasma chamber and 0.03 Pa in the deposition chamber. The lowest pressure reached in the deposition chamber was about 10^−5^ Pa when no gas was injected. The deposition chamber was provided with a 0.1 mm diameter circular orifice connecting it to the quadrupole mass spectrometer for sampling the gas of interest. The turbo molecular and rotary pumps ensured sufficient purity conditions in the chamber required by the spectrometry diagnostics by keeping the instrument pressure below 10^−4^ Pa.

The inlets for the gas and the precursor were located on one side of the plasma chamber that was opposite to the deposition chamber, as shown in Figure 1. Argon and oxygen were injected using two separate mass-flow controllers. The corresponding gas mixture could be considered to be found in a well-mixed state because the gas diffusion speed, 1.26 m/s, exceeded the gas flow speed, 0.04 m/s. The gas expansion by the sonic nozzle generated a focused and supersonic jet enabling the deposition at high grow rates [44]. As a result of the substantial volume increase, the temperature, pressure, and density of the transported material decreased following the isentropic law, in which the gas particles are accelerated and the Mach number (the ratio between the velocity of a particle and the local sound speed) takes values larger than 1.

Part of the NP thermal energy was transformed into a well-oriented beam of matter that started forming a supersonic jet. The expansion ended at the Mach disk, a position along the flow trajectory where a normal shock occurred. At the Mach disk, the values of the temperature, pressure, and density of the gas reached the background values and became subsonic (Mach number drops below one). The properties of the supersonic beam largely depended on the size and shape of the nozzle, in addition to the thermodynamic properties of the gas [45,46]. The geometry of the supersonic jet is represented in Figure 1c.

The supersonic regime occurred for distances $z<z_{M}$, where $z_{M}$ represents the position of the Mach disk from the nozzle and is given by,

(1) $$z_{M}=0.67\;D\;\sqrt{R}$$

where *D* is the diameter of the circular orifice at the nozzle and $R=p_{P}/p_{D}$ is the ratio between the pressures at the plasma and deposition chambers, respectively. In our experiments, we used a diameter *D* = 6.9 mm, and we used Equation (1) to find that R fell in the range of 2 < *R* < 40 for $z_{M}$ changing between 6.5 and 29 mm, depending on the background pressure we set in the deposition chamber. The particle density in the jet, *n*(*z*) at position *z*, could be estimated from the empirical relation [47]:

*n*(*z*) = *n*_0_ [1.44 (*z*/*D*)^2^ − 0.65 (*z*/*D*) + 0.87] ^−1/*γ*^ (2)

where *n*_0_ is the gas density inside the plasma chamber and γ is the adiabatic index of the gas.

RF plasma sources are usually employed to dissociate the precursors, and in the case of inductively coupled plasma (ICP) sources, they can provide uniform and high charge densities along with low ion energies that can be accurately controlled in most cases [43]. Here, the plasma was generated by a 13.56 MHz radiofrequency power generator (Huttinger PFG 1600 RF, Schwaig bei Nuremberg, Germany) connected by a tunable matching box. By feeding the RF antenna with a 450 W input power, an inductively coupled discharge and a stable dense plasma could be generated [44,47]. The plasma was analyzed by optical emission spectroscopy, Langmuir probes, and I–V measurements. The electron temperatures were about 1 eV, and the plasma densities were between 10^11^ and 10^13^ cm^−3^ depending on the reactor parameters.

We let the titanium precursor enter the plasma chamber after the argon/oxygen ratio had been adjusted so that it yielded an inductively sustained plasma discharge at the input power of 450 W. We used titanium (IV) tetra–isopropoxide Ti(OCH(CH_3_)_2_)_4_ (atomic mass M = 284 amu). At 20 °C, the precursor was a liquid. Once it was heated, it reached its vapor phase. As a rule, we used a power transformer for this purpose. The gas temperature could be varied to produce different precursor flows from 0.25 to 0.8 g/h, as calculated by the resulting precursor consumption during deposition process, and was monitored using a thermocouple located above the precursor tank. By heating the TTIP precursor between 40 and 50 °C, a steady state precursor flow could be created in the plasma chamber.

We employed the quadrupole mass spectrometer (QMS) Hiden EQP-1000 Analyzer (Warrington, UK) in order to detect neutrals, radicals, and ion species [42]. The QMS could be positioned along the jet center line (the *z*-axis), so we are able to sample chemical species at different positions, as well as provide in situ real-time sampling.

### 2.2. Thin Films Characterization

The deposition was performed varying the distance between 5 and 30 mm inside the deposition chamber. Slabs of oxidized single crystal silicon were employed. The substrates were firstly cleaned using pure ethanol. An aluminum mask on the substrate defined the contour of the deposition area of 7 × 7 mm^2^. The deposited films were annealed at 500 °C for about 20 min to remove any remaining organic impurities. The thickness of the PA-SJD films was evaluated with a Dektak 8 Stylus Profilometer from Veeco (Plainview, NY, USA). The applied stylus tracking force was 15 mg, and its nominal vertical resolution was 1 Å. To characterize the surface morphology, the sample surface was scanned with a P47-PRO NT-MDT AFM (Moscow, Russia) working in semi-contact mode using a silicon tip. The resonance frequency was 245 kHz, and the constant applied force was 12 N/m. The tip had a curvature radius of less than 10 nm. The resolution employed in each scan was fixed at 256 × 256 points [45,47]. To collect secondary electrons, the in-lens ZEISS 1530 SEM detector (Oberkochen, Germany), equipped with a Schottky emitter and operating at 10 keV, was used for the microscopic analysis of the deposited thin films’ morphology. In previous works, this task also involved TEM imaging, and we will refer to some of those analyses performed in our previous papers [32,33]. We analyzed the chemical composition of the annealed thin film by means of RAMAN spectroscopy using a Labram (Dilor—JobinYvon, HORIBA, Kyoto, Japan) device, X-ray diffraction (XRD) spectroscopy (Rigaku SmartLab, Rigaku Corporation, Tokyo, Japan), and Fourier Transform infrared spectroscopy (FTIR) (Nicolet iS10, Thermo Fisher Scientific, Waltham, Massachusetts, USA).

## 3. Results and Discussion

### 3.1. Plasma-Assisted Supersonic Jet Characterization

We set up a pressure of 10 Pa in the first chamber to ignite the plasma using a 2:3 mixture of Ar and O_2_, respectively. The reason for this is that argon allowed us to stabilize the plasma and oxygen ensured the dissociation of the precursor. Above 175 W of input power, the discharge coupling changed from capacitive to inductive, which was of interest to us. Thus, most of experiments were performed at 450 W power.

**Figure 2:**
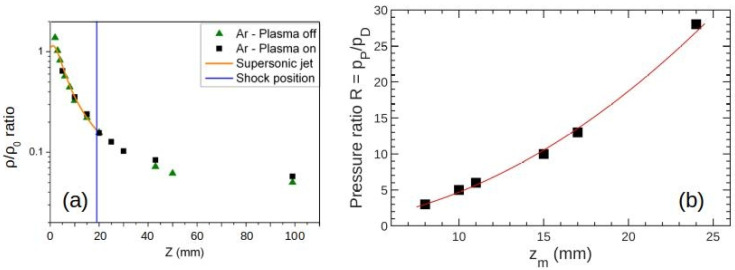
(**a**) Normalized argon number density, ρ/ρ_0_, as a function of position *z* (mm), measured in the deposition chamber for a pressure of 8 Pa in the plasma chamber, yielding *R* = 28. The Mach disk positions $z_{M}$ (Equation (1)) are indicated by the blue vertical bar. (**b**) *R* vs. $z_{M}$. The red continuous line is a parabolic fit, *R*∼$z_{M}^{2}$ from Equation (1) that is in good agreement with the experimental data.

By moving the QMS along the jet axis, we were able to detect a signal (measured in counts per seconds) proportional to the density of species. For each measured mass, the acquired data were renormalized to fit the density ratio expected from Equation (2). The statistical errors were within 1%. As can be seen in Figure 2, the isentropic expansion law, as given by Equation (2), yielded results in good agreement with our experimental data for distances *z* smaller than the Mach disk value.

In Figure 2b, we report $z_{M}$ for different pressure ratios, 3 < *R* < 28, all used in the same plasma chamber pressure, $p_{P}$ = 8 Pa. The different *R* values were obtained by varying the pressure $p_{D}$ in the deposition chamber up to 4 Pa. The Mach disk position $z_{M}$ increased when increasing *R*, according to Equation (1). When the precursor was introduced inside the plasma, their reactive species (such as oxygen, the electrons, and the heating produced by chemical oxidations) promoted the formation of several organic and organo–metallic chemical groups.

**Figure 3:**
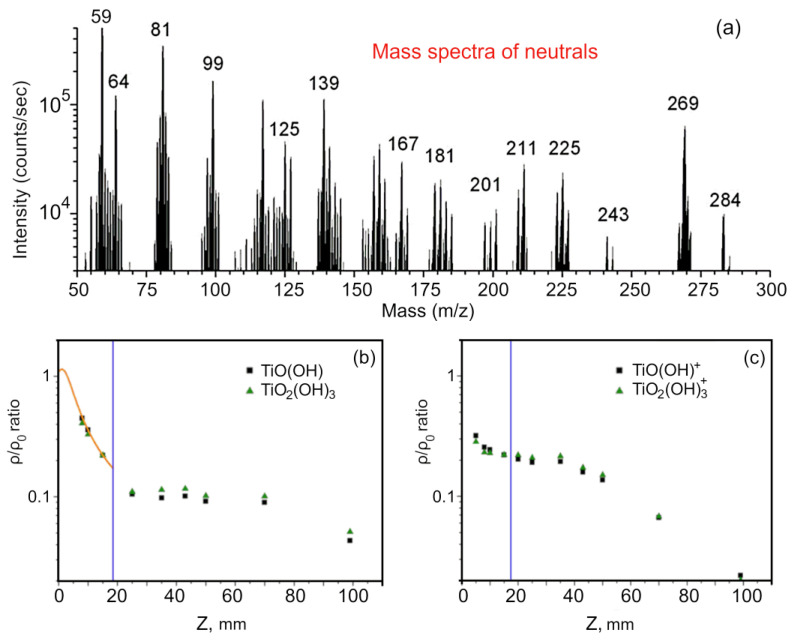
Generated species in the jet for 8 Pa of pressure in the plasma chamber. (**a**) Mass spectra (for masses between 50 and 300 amu) of precursor species in the discharge at 5 mm from the nozzle. (**b**) Normalized number density, ρ/ρ_0_, vs. *z* (mm) of the two main TiOx species for *R* = 13 along the jet. (**c**) Normalized number density, ρ/ρ_0_, vs. *z* (mm) for ion species produced by plasma flowing along the jet at *R* = 13. The Mach disk positions $z_{M}$ are indicated by blue vertical bars.

In Figure 3a and Table 1, the main precursor species, together with their masses in amu, are reported. Larger molecule clusters in the plasma and deposition chambers could be considered negligible. The TTIP dissociation products could be easily identified, of which the most abundant heavy molecules were TiO_x_H_y_ seeds that provided the mass peaks at 81 and 99 amu.

Neutrals scans were performed during the plasma generation. The QMS counts along the *z* axis are reported in Figure 3b,c, which shows the peak area of some gas species calculated from mass spectra acquired at different positions for *R* = 13. In particular, densities of neutral TiO_2_H and TiO_3_H_3_ seeds are reported along the jet axis in Figure 3b. The precursor seeds followed the theoretical isentropic expansion law, but, in contrast to the light gas carriers, their behavior was different after the Mach disk location: the measured signal did not decrease. The peak area for the products of the precursor dissociation was constant after the Mach disk location. This phenomenon may have been due to the longer mean free path related to their heavier masses. Due to their inertia, the TiO_X_ seeds did not expand further after the Mach disk location. In particular, different accelerations due to inertia promoted particle separation and the retention of heavier species along its centerline. This phenomenon, due to an inertial effect that maintained the heavier species near the jet centerline, was particularly interesting because it allowed us to perform thin film deposition far away from the nozzle, where the jet was no longer supersonic. Similar results were found at a different pressure ratio. Figure 3c reports a similar trend for the ionized TiO_x_ seeds along the plasma jet, although their density decreased faster after 50 mm from the nozzle, probably due to neutral recombination [48].

### 3.2. TiO_2_ Thin Film Characterization

PA-SJD was able to produce the depositions under the same experimental conditions. However, even if the precursor temperature was the same in different experiments, the TTIP mass flow could have changed inside the plasma chamber. The deposition rate could decrease after a set of successive depositions because the residual precursor could choke the chopper tube of the injector. However, repeatability was ensured when, after one or two hours of operation, the precursor injection system was refilled and cleaned up.

The film chemistry was controlled by tuning the plasma parameters such as plasma power and gas mixture. The determination of the optimal stoichiometry of TiO_2_ films by varying the concentration of oxygen, argon, and precursor mixture was the subject of a preliminary study. Here, we considered the effects of heating the organic precursor mixture in the plasma chamber at different temperatures. Above 60 °C, high precursor fluxes could be obtained, but the precursor was not effectively dissociated, and we detected the presence of organic residues even after annealing. At lower temperatures between 40 and 50 °C, however, he precursor was efficiently dissociated and titanium oxide seeds, mainly Ti-like light species, were transported by the supersonic jet towards the substrate and yielded TiO_2_ quasi-stoichiometric films, as shown below by the film analysis.

**Figure 4:**
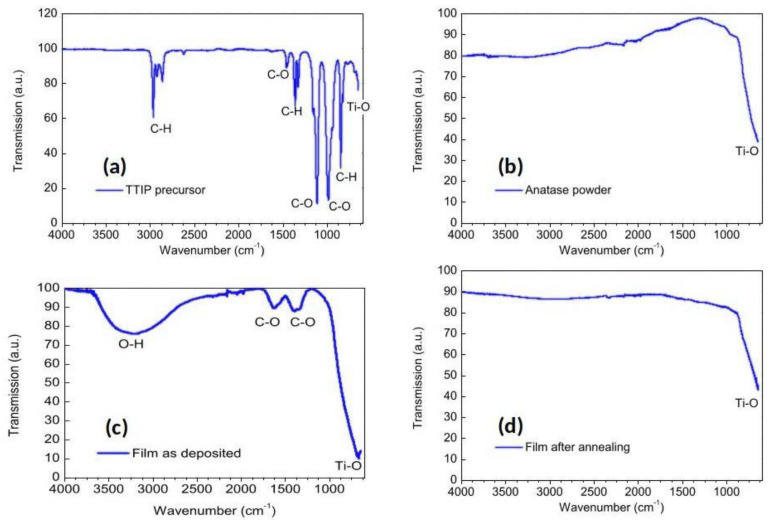
FTIR spectrum of: (**a**) TTIP precursor, (**b**) anatase powder, and (**c**) the as-deposited film, (**d**) annealed film. The spectrum of as-prepared film does not feature C–H stretching, indicating that the plasma efficiently dissociated and oxidized the precursor. The spectrum of annealing film (**d**) is similar to that of pure anatase (**b**).

The chemical composition of the produced film was analyzed by means of FTIR spectroscopy. Figure 4 reports the FTIR spectrum of the titanium precursor.

The spectra shown in Figure 4a reveal the presence of several chemical bonds of the TTIP precursor (Ti(OCH(CH_3_)_2_)_4_). Ti–O stretching can be identified around 635 cm^−1^ (in literature the value is 626 cm^−1^). The peaks at 2864, 2960, and 2924 cm^−1^ represent C–H stretching bonds. In addition, traces of C$H_{3}$ bending are found at 1366, 1318, and 844 cm^−1^. To be noted is that C–O stretching can be identified at 1114 and 982 cm^−1^, and C–O vibrations are represented by the peak at 1450 cm^−1^.

A typical spectrum measured on the deposited film is reported in Figure 4b. The spectrum is completely changed with respect the TTIP one reported in Figure 4a. In particular, we could not detect C–H stretching, indicating that the plasma efficiently dissociated and oxidized the precursor. The two further broad peaks at 1637 and 1380 cm^−1^ are related to C–O-conjugated vibrations typical of transition metals. The last detected peak is the one corresponding to Ti–O stretching, shifted to 700 cm^−1^ because of the attachment of organic molecules. Then, we could observe O–H stretching vibrations between 3000 and 3500 cm^−1^. We can conclude that CH chemical groups were missing in the as-deposited film, while some organics (C–O and O–H) were still present. 

Figure 4c reports the analysis of a powder of pure anatase TiO_2_. In this case, we detected only one peak near 650 cm^−1^ that corresponded to Ti–O stretching. In Figure 4d, a spectrum similar to Figure 4c was obtained for the annealed film at 500 °C. Here, the spectrum reveals an only peak near 650 cm^−1^ related to Ti–O stretching. This analysis shows that the annealing was able to promote the purity of TiO_2_ films, removing organic residuals. Additionally, annealing is essential to vary the crystallinity of deposited films, as we show later with Raman spectroscopy.

**Figure 5:**
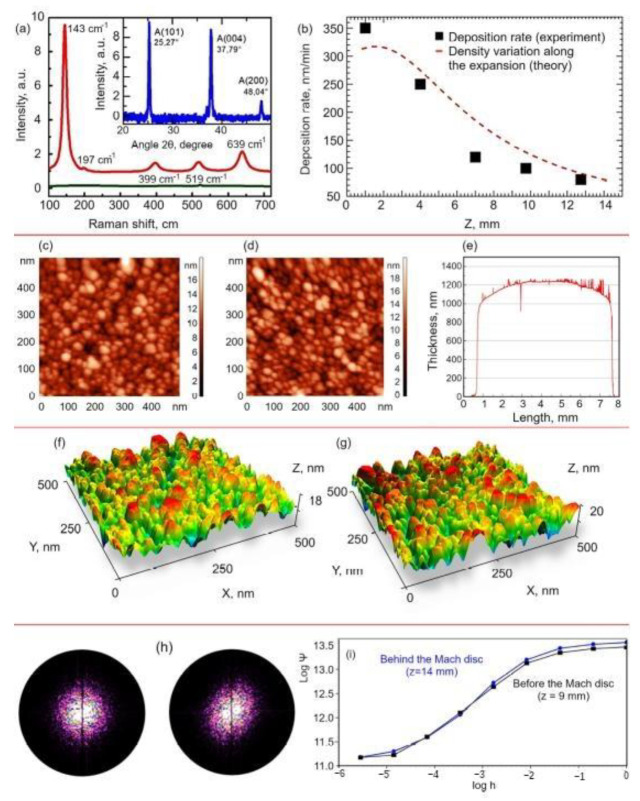
(**a**) Raman spectra (red line) following annealing processing at 500 °C. Characteristic peaks of anatase are labeled with the letter A. Raman spectrum before annealing (green line) shows that the as-deposited film spectrum is amorphous. In the inset, the XRD spectrum is shown (blue lines) (**b**) Experimental deposition rate (nm/min) (squares) compared to the density variation expected from Equation (2) (dashed line). (**c**,**d**) AFM images obtained using by scanning an area of 500 nm × 500 nm at distances of *z* = 9 mm and *z* = 14 mm. (**e**) Film thickness profile of the sample located at *z* = 14 mm. The precursor flux was 0.8 g/h and R = 10 for both samples. (**f**,**g**) Three-dimensional reconstruction of the nanostructures. (**h**) Power spectra density for the samples deposited at *z* = 9 mm (left) and *z* = 14 mm (right); the first sample demonstrated higher symmetry. (**i**) Distribution of fractal dimensions for the two samples. The sample deposited behind the Mach disk demonstrated slightly higher fractality, i.e., more developed nanostructured surface.

Figure 5a shows a typical result of a Raman spectroscopy experiment performed on the as-deposited and the annealed films. The Raman spectrum of the as-deposited film clearly suggests that we were dealing with an amorphous material. The Raman spectrum, following annealing at 500 °C for 20 min, shows a peak at 143 cm^−1^ and three mid-intensity peaks at 399, 519, and 639 cm^−1^ that are the signatures of the complete transformation of the film into anatase TiO_2_ [49].

XRD analyses were conducted on annealed film samples to promote the formation and coalescence of crystalline domains. In the top box of Figure 5a, the diffraction spectrum measured on a deposit from TTIP under optimized conditions after annealing is shown. A comparison with the literature data, collected in a dedicated database available online [50], showed that the film was made of titanium dioxide in the form of allotropic anatase. We can therefore conclude by saying that heat treatment promoted the transition of our deposit into the allotropic anatase form. In fact, there were peaks due to scattering at angles of 25°, 38°, 38°, and 48° corresponding to reflection from some of the crystalline planes of anatase. Conversely, no angle corresponding to the crystalline phases of rutile or brookite appeared in the XRD spectrum. The absence of prominent shifts in the intensity and position of diffusion angles was also significant because it ensured that: (1) there was no residual stress in the film remaining during film growth, (2) there were no significant stoichiometry defects (for example, a sub-stoichiometry in oxygen would induce structural changes in the size of the crystalline cell), and (3) the presence of elements (both substitutional and interstitial) with different atomic radius from Ti and O was negligible so Ti and O occupied the positions of equilibrium of the anatase form. Finally, by applying the Debye–Scherrer formula [51] to the peaks shown in the box of Figure 5a, the size of the crystalline grains in the deposited film could be estimated. Using a form factor of 0.8 and an FWHM width of 0.31, the grain size could be estimated in the order of 25 nm.

We used a mechanical profilometer to determine the thicknesses and deposition rates of the samples. The growth rate was measured using the average height of the film divided by the film deposition time. Different growth rates were observed for depositions performed at different positions along the jet axis, which followed the gas expansion law, as shown in Figure 5b. The deposition rate was reported as a function of distance from the nozzle *z* for a given constant TTIP precursor flux. The theoretical gas density variation along the expansion, deduced from Equation (2), was applied the experimental data. The comparison showed that the growth rate of TiO_2_ thin films was related to the abundance of TiO_x_ seeds, as expected for an isentropic expansion. Specifically, the deposition rate can be varied, from few tens nm/min to more than 300 nm/min, depending on the substrate position from the nozzle. The deposition rates can widely vary between some tens of nm/min up to tens of µm/min as a function of the precursor flows controlled by the heating temperature, allowing one to use the technology for different purposes [33,36].

By annealing at 500 °C for 20 min, the film thickness decreased by less than 10% compared to the as-deposited film. In Table 2, the thicknesses of the as-deposited and the annealed films are listed as a function of the precursor heating temperature and time deposition. At higher annealing temperatures, the thickness greatly decreased by up to 50%.

AFM images, obtained at two different distances from the nozzle before the Mach disk (*z* = 9 mm and *z* = 14 mm), are reported in Figure 5c,d on the scale of hundred nanometers. Grain clusters at the film surfaces attained similar sizes of about tens of nanometers. The two film depositions were performed with the same precursor flow and R = 10 so that we could speculate that collisional processes were negligible in the supersonic jet and mostly occurred in the plasma chamber, and nanoclusters mainly assembled in the plasma phase. This shows that before the Mach cone, the collisional rate was very low, thus allowing us to control the grain size of nanoparticles by tuning the pressure in the plasma chamber. A similar result was reported in literature when aerosol nanoparticles were supersonically accelerated [34]; it was found that the grain sizes embedded in the thin films were similar to those of primary aerosol nanoparticles, i.e., nanoparticles were conserving their sizes during the acceleration. The general observation deduced by experiments and simulations involving supersonic aerosol jets of similar energies as those here, (0.1–1) eV, is that sizes for primary nanoparticles and crystal grains in film are conserved.

By exploiting the fact that particle speed in the jet abruptly decreased at the Mach disk location and grew slower at larger distances from it, it was possible to intercept the moving species at those large distances from the nozzle by just moving the position of the deposition substrate. Furthermore, since the jet had a transversal profile near the Mach disk that ensured quite flat deposition profiles, the resulting TiO_2_-deposited films displayed a rather good spatial uniformity in the transversal direction of up to 7 mm in comparison to the deposition performed at *z* = 14 mm. In Figure 5e, the deposition rate was about 62 nm/min. A specific discussion on the film thickness uniformity was described in a previous paper [30].

Figure 5h,i illustrates the results of a fractal analysis of the fabricated nanostructures. The power spectra density shown in Figure 5h are for the samples deposited at *z* = 9 mm (left, before the Mach disc) and *z* = 14 mm (right, behind the Mach disc). The first sample demonstrated higher symmetry. Figure 5i shows the distribution of fractal dimensions Ψ for the two samples as a function of height h. The sample deposited behind the Mach disk demonstrated a slightly higher fractality, i.e., more developed nanostructured surface.

**Figure 6:**
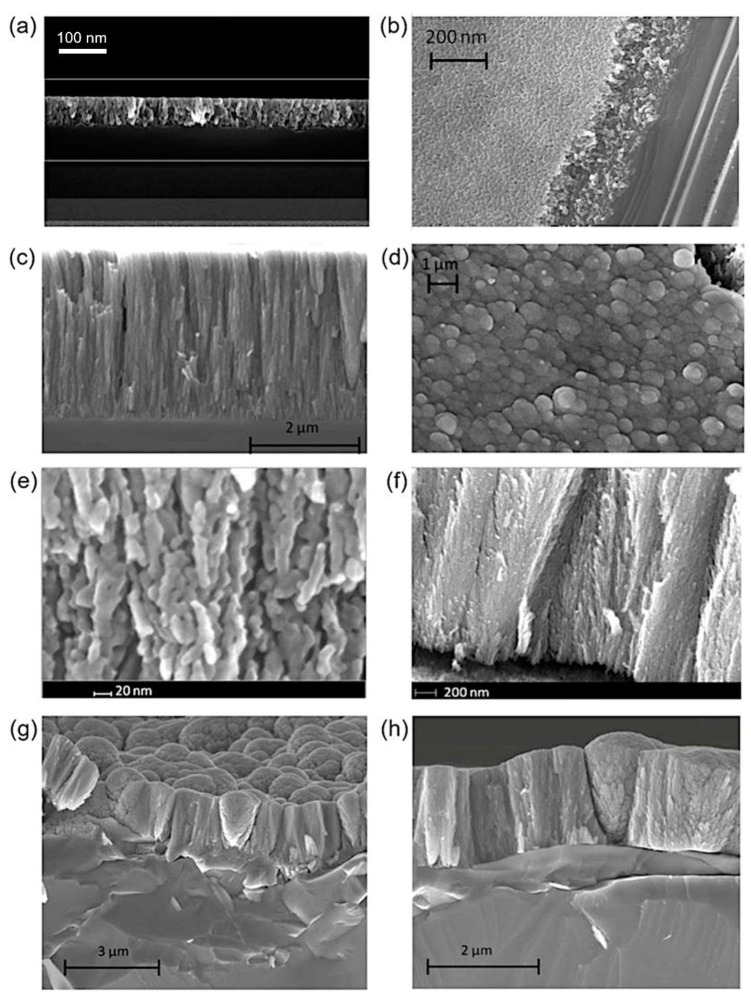
SEM analyses of annealed samples. (**a**,**b**) Cross-section and top view SEM images of a film deposited for 10 min located at *z* = 10 mm. (**c**,**d**) SEM images of a film deposited for 40 min located at *z* = 10 mm. Cross-section and top view. (**e**,**f**) SEM images of a film deposited for 30 min located at *z* = 10 mm. In (**a**–**f**), R = 10. (**g**,**h**) SEM images of a film deposited for 30 min located at *z* = 10 mm for and R = 6 (cross-sections at different magnifications). The precursor flow was 0.8 g/h.

While the grain size could be controlled with either the plasma pressure or by locating the substrate at distances far away from the Mach disk, the resulting morphology could still be affected by the R parameter. In particular, R determines the morphology of the incipient tree-like structure of a thin film. Before analyzing the role of R, we discuss the structural assembly of a thin TiO_2_ film. SEM images of a typical annealed thin film are reported in Figure 6a,b for R = 10 and fixed TTIP flow at 0.8 g/h at *z* = 10 mm. In Figure 6a, the cross-section of the film shows nanoclusters mostly packed along the vertical growth on 100 nm. The top view of Figure 6b suggests a relatively smooth surface where the tips of rods emerged and were grouped to form structures composed of a few assembled nanoclusters.

The properties of the thus-produced thin films are reproducible. Similar nano-assembled structures, composed of few nanoclusters and having rod-like and weakly disordered elongated-grape shapes, can be fabricated when varying R. Thicker films develop different hierarchical structures as a function of the R parameter. For R = 13, an annealed film obtained at longer deposition times (in this case, after 40 min) and located at the same position (*z* = 10 mm) showed an elongated compact structure, as reported in Figure 6c,d. The vertical growth corresponded to compact films with a relatively low porosity of about 3 µm thickness. A previous study [30] showed that these films displayed a high porosity in percentage of voids of between 35% and 45%. The surface of the film (see Figure 6d) showed emerging, well-packed, tree-like structures. After decreasing R, the tree-like nanostructures opened up, showing a more complex type of assembly. In Figure 6e,f, the cross-section of the annealed film, deposited at R = 10, shows an increase in the transversal direction, that is, the tree-like hierarchical assembly fanned out. The size of nanoclusters was of the order of 10 nm, and their tree structure could be better visualized by a TEM analysis [36]. The latter showed the formation of a zig-zag-like structure composed of single, mostly crystalline, domains of 10 nm in size.

Films of TiO_2_ with this hierarchical structural morphology are characterized by a large surface area and have been fabricated as photocathode for photovoltaic applications [36]. In fact, it has been demonstrated that high-surface-area nanostructures enhance sunlight and dye absorption in specifically designed solar cells, known as dye-sensitized solar cells, and ordered nanostructured networks offer a more direct charge transport pathway that improves the same electron mobility. Similar brush-like structures, which are mostly vertically oriented on films, have been fabricated with PLD. In this case, they are well-separated from each other and become more open as height increases. The enhanced surface area of the emerging structures does not seem to result into an overall larger surface area due to the presence of large voids developing in between neighboring structures and to the larger spacing among the growing branches [52].

At lower values, *R* = 6, the structures in our study also developed conical shapes, as shown in Figure 6g,h. Conical and squared shapes alternated in the film, exploiting the available free space for growth. We can imagine that tree trunks were formed from the first rod elements and that they grew while leaning against each other to merge into larger structures. In Figure 6g,h, it is also possible to appreciate the presence of leaves attached to the structures. From above, the surface looks like a coniferous forest similar to a cauliflower. We can argue that the effect of the ballistic deposition depended on the energy and shape of the supersonic jet of seed nanoclusters, which was turn regulated by the parameter *R*. 

At high *R*, the jet was well-oriented and the fast clusters attached themselves to the surface on top of previously deposited clusters. At low *R*, the jet was less energetic and less oriented and the clusters had transversal velocity that allowed them to laterally attach to the vertical structures, thus forming branches and leaves. In previous experiments, with supersonic aerosol jets [34], a decrease in deposition energy promoted less densification and no hierarchical grain growth, though with similar film mass densities of ∼60% of bulk (i.e., about 40% of void densities) than ours. By means of PLD [52], porosity has been controlled by tuning the pressure in a vacuum chamber to between 20 and 500 mTorr. In [35], nanoporous films were grown by varying the background gas pressure in order to control the nature of the species on the film, their kinetic energy, and their aggregation dynamics. 

**Figure 7:**
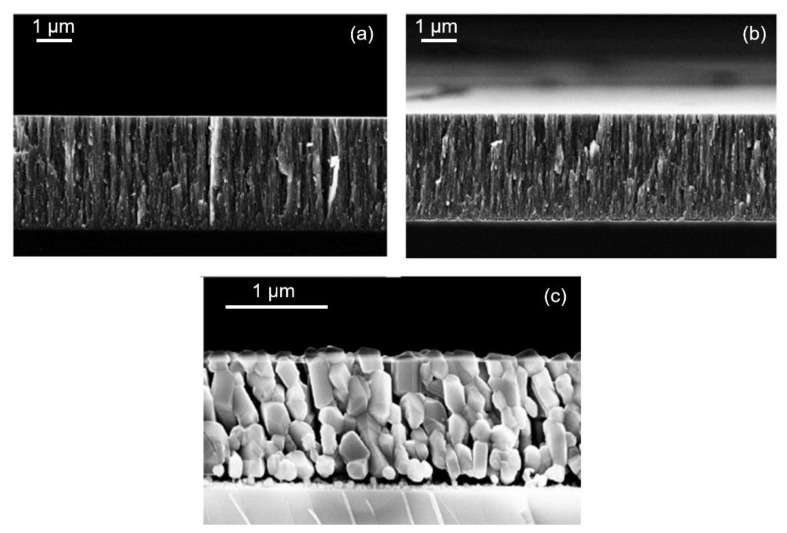
SEM images for a film deposited on a substrate at *z* = 8.4 mm, a precursor temperature of 51 °C, a deposition time of 30 min, and R = 6 (last sample reported in Table 2). (**a**) As-deposited film (thickness of 5800 nm); (**b**) annealed film at 500 °C for 20 min; (**c**) annealed film at 1000 °C for 60 min.

Finally, we briefly discuss the effects of the annealing process on the morphology of films. In Figure 7a,b, the SEM images of an as-deposited film and its annealed form at 500 °C show that the appearance at the nanoscale of the structures remained unaltered and that the thickness of the annealed film was slightly decreased by about a 2% with respect to the as-deposited film. On the contrary, when the film was annealed at the higher temperature of 1000 °C, as can be seen in Figure 7c, its nanostructure completely changed into a rutile one and its thickness decreased by about a 30%.

### 3.3. TiO_2_ Films on Wider Areas

In order to deposit films over wider areas, tests were performed by gradually vertically moving the substrate by few millimeters (generally 5 mm) at a time step of 2–4 min. The nozzle’s ending orifice was replaced by a larger slit whose height and width (20 and 8 mm, respectively) were set to provide a conductance similar to the one of the circular orifices. A small slab made of glass coated by a fluorine-doped tin oxide was used as the substrate, which was fixed to a movable vertical bar. 

**Figure 8:**
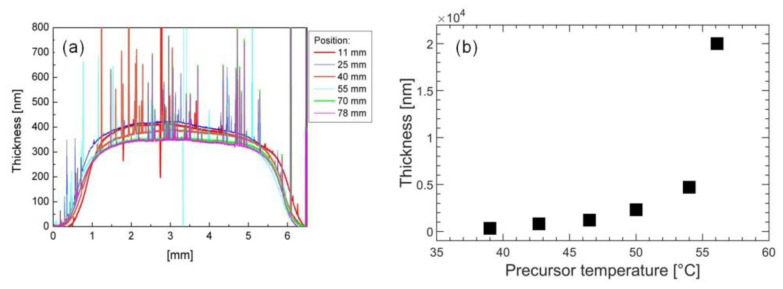
(**a**) Profilometer scans performed at different *x* positions (0 < *x* < 5 mm) on an area of 5 × 80 mm^2^ of TiO_2_ thin film. The considered *y* positions (0 < *y* < 80 mm) are shown in the inset. The thin film was deposited at a low deposition rate at 6 mm from the nozzle slit while vertically moving the substrate back and forth by 5 mm every 2 min. (**b**) Thickness of the thin films vs. TTIP temperature obtained at *z* = 9 mm.

The deposition was repeated by moving the substrate back and forth a few times (depending on the expected film thickness). The mean distance of the substrate from the nozzle was 7 mm. Figure 8a shows the result for an 80 × 5 mm^2^ thin film, which was exposed to the seeded jet for few minutes at 41 °C with a small precursor flow. The profilometer scans refer to different sample lines along the sample minor axis of 5 mm. The whole area was successfully covered with a nearly uniform TiO_2_ nanostructured deposition with an average height of 350 nm. At the higher precursor temperature of 50 °C, it was possible to boost the deposition rate and obtain a film with a thickness of the order of few μm.

By increasing the precursor temperature, larger precursor fluxes could be injected in the plasma chamber. The relation between temperature and precursor flux appeared linear, and it could be studied by looking at the dissociation products by QMS. In Figure 8b, the trend of the thickness is plotted as a function of temperature at a fixed position from the nozzle, *z* = 9 mm. We found that the growth rate was almost linearly dependent on the precursor temperature in the range from 40 to 50 °C.

Along these lines, another important feature concerns the role of the precursor flux. In the above discussion, we highlighted that the grain size in the Mach cone was conserved. The grain size could instead sensitively depend on the applied precursor density in the plasma chamber. As the precursor flux increased, the metal oxide aggregation phenomena increased due to the higher collision rates inside the plasma chambers. One can conclude that in the Mach cone, the dimension of the TiO_2_ seeds was conserved, while the grain size could be modified by changing the precursor flux injected in the plasma chamber. Thus, by increasing the precursor flux, two effects were produced: an increase in the grain size in the plasma chamber and an increase in the concentration of clusters inside the jet. Both yielded an increase in film deposition rates. 

## 4. Conclusions

In PA-SJD, clusters of molecules and nanoparticles are formed in inductively coupled plasma, then transported in the expanding jet, and deposited on a substrate. In the former, the employed argon–oxygen plasma produces a reactive environment where an organo–metallic precursor can become well-dissociated and oxidized. Depending on the jet parameters, different nanostructure sizes and deposition rates can be achieved. We considered TiO_2_ nanoparticles as seeds of thin films, whose structure and morphology were analyzed and characterized.

The titanium nanoparticles present in the jet were identified using QMS measurements. By sampling neutral gas species along the supersonic jet, we studied their profiles. Many molecules of partly dissociated precursors could be observed. The study of the supersonic jet axis profiles for gas carrier and the TiO_x_ seeds showed that titanium nanoparticles followed an isentropic expansion, like that of the light gas carrier, until the Mach disk location. After the Mach cone, titanium nanoparticles maintained their density over long distances due to an inertial effect that kept the heavier species near the jet centerline.

Nanostructured TiO_2_ thin films deposited by PA-SJD could be grown at different deposition rates depending on the precursor mass flow and the distance of the deposited film from the nozzle. A thermal annealing performed at 500 °C could successfully remove any organic impurities while also setting the crystalline structure of the TiO_2_ to anatase, which is the most interesting for most applications.

The growth rate ranged from few nm/min to few hundreds nm/min and scales with the isentropic density relation along the supersonic jet. Depositions were also performed over wide areas of the order of cm^2^ using a movable substrate and changing the dimension of the supersonic nozzle. The morphology of the thin films was characterized by SEM imaging. The SEM images of some selected samples confirmed a vertical nanostructure with grain diameters of about 15 nm. Experiments also confirmed that the precursor density used inside the reactor chamber determined the grain size of the thin film building blocks. Depending on the selected supersonic jet parameters, different hierarchically complex nanostructures can be deposited in a controlled fashion, thus yielding structures with a predetermined desired morphology.

Despite the above-mentioned advantages of the technology based on supersonic plasma jets, scaling up towards industrial applications may represent a problem. Though any low-pressure system has inherent challenges regarding integration and scale-up, solutions based on plasma sources of larger volume to increase the processed surface area, along with deposition systems employing supersonic multi-jet systems and systems with moving stages, could be adapted to the industrial scale in order to keep the definite advantages of the plasma treatment such as control over the deposition, additional possibilities in morphology, and material structure control via energetic particles in plasma.

## Figures and Tables

**Table 1:** Main species of the TTIP dissociation measured during experiments and corresponding mass M (amu).

M	Species	M	Species
15	CH_3_	139	TiO_2_(OCH(CH_3_)_2_)
43	CH(CH_3_)_2_	167	Ti(OCH(CH_3_)_2_)_2_H
59	OCH(CH_3_)_2_	181	TiO(OCH(CH_3_)_2_)_2_–H
64	TiO	211	Ti(OCH(CH_3_)_2_)_3_H–CH_3_
81	TiO_2_H	225	Ti(OCH(CH_3_)_2_)_3_
99	TiO_3_H_3_	243	Ti(OCH(CH_3_)_2_)_4_–CH(CH_3_)_2_
125	TiO(OCH(CH_3_)_2_)	269	Ti(OCH(CH_3_)_2_)_4_–CH_3_

**Table 2:** Film thickness versus temperature T (°C) of precursor (precursor flows) for the as deposited and annealed films.

Precursor T (°C)	Thickness as-Deposited (nm)	Thickness after Annealing (nm)		Deposition Time (min)
		at 500 °C	at 1000 °C	
40.5	350	330	-	15
46.5	1200	1100	700	
51.5	3000	2900	1500	
56.1	20,000	19,500	15,000	
51	5800	5700	1800	30
